# Innovative liposomal coumarin: A promising solution for enhancing soft white cheese quality

**DOI:** 10.1371/journal.pone.0315771

**Published:** 2025-03-18

**Authors:** Salameh Alqaraleh, Laila Al-Omari, Ghadeer Mehyar, Moath Alqaraleh, Walhan Alshaer, Hiba Abdelnabi, Sarah Jaradat

**Affiliations:** 1 Department of Nutrition and Food Science, The University of Jordan, Amman, Jordan; 2 Department of Medical Laboratory Sciences, Al-Ahliyya Amman University, Amman Jordan; 3 Department of Medical Laboratory Sciences, Al-Balqa Applied University, Al-Salt, Jordan; 4 Cell Therapy Center, The University of Jordan, Amman, Jordan; University of Michigan, EGYPT

## Abstract

**Background:**

Coumarin is a natural bioactive compound found in many plants and acquires antibacterial, antioxidant and anticoagulant activities. The antibacterial activity of coumarin has never been tested after being encapsulated in liposomes. This study was carried out to screen the main functional components of *Rubus canescens* DC crude extract (CE), develop a coumarin nanoliposome, and test its anti-bacterial and antioxidant properties.

**Methods:**

*R. canescens* DC CE was screened for its main functional compounds using liquid chromatography-mass spectrometry (LC-MS). Pure commercial coumarin was loaded into liposomes and characterized in terms of surface morphology, hydrodynamic diameter, zeta potential, and encapsulation efficiency (EE). The antioxidant activity of coumarin was evaluated against ascorbic acid. The antibacterial activity of both coumarin alone and liposome-encapsulated coumarin against *Staphylococcus aureus*, *Salmonella typhimurium*, and *Pseudomonas aeruginosa* inoculated in soft white cheese (SWC) was also evaluated.

**Results:**

The predominant natural constituent of *R. canescens* DC CE, was coumarin. Comparing the DPPH scavenging activity of coumarin to that of ascorbic acid, coumarin exhibited an insignificant effect (p ≥ 0.05). The minimum inhibitory concentration (MIC) values for coumarin against *P. aeruginosa*, *E. coli*, *Staph. aureus*, *S. typhimurium*, and *L. monocytogenes* were 2.5, 2.5, 2.5, 1.25, and 1.25 µg/ml, respectively. The minimum bactericidal concentration (MBC) values for coumarin against these microorganisms were 5, 5, 5, 2.5, and 2.5 µg/ml, respectively. Coumarin was successfully loaded into nanoliposomes, which had a polydispersity index (PDI) of 0.36 ±  0.35 Đ, hydrodynamic diameter of 127.8 ±  0.3 nm, zeta-potential of -61.03 ±  2.9 mV, and EE of 40.93 ±  0.2%. Both the coumarin alone and the liposome loaded with coumarin showed antibacterial effects against the inoculated bacterial strains in SWC over a 30-day storage period at 4^°^C.

**Conclusions:**

Coumarin was successfully formulated into a nanoliposome, and showed antibacterial activity against *P. aeruginosa*, *S. aureus*, and *S. typhimurium*.

## Introduction

Over the past few years, there has been a notable trend favoring the adoption of natural food additives over synthetic counterparts, especially emphasizing the utilization of phytocompounds sourced from plants. These substances are recognized for their ability to improve the safety and quality of food items by inhibiting microbial activity and oxidation reactions [1].

In pursuit of this goal, there has been a growing fascination with formulating functional ingredients encapsulated in liposomes for incorporation into food products, aiming to enhance safety, quality, and nutritional content. These functional ingredients encompass plant-derived phenolic chemicals, probiotics, and prebiotics, as well as peptides, colostrums, egg yolks, and others. Their thorough investigation is attributed to their enhanced taste, ready accessibility, prolonged shelf life, and cost-effectiveness [2]. Moreover, nanotechnology holds the promise of transforming food systems, as ingredients at the nanoscale can enhance the functionality and bioavailability of specific nutrients like vitamins and minerals. This is achieved through encapsulation, enabling more efficient delivery and absorption by the body [3]. In Jordanian rural areas, over 2,500 plant species from 142 families and 868 genera have been identified, with more than half of them having medicinal properties [4]. *Rubus canescens DC* is commonly found in the rural areas of southern Jordan and is known to contain ingredients exhibiting potent antimicrobial and antioxidant properties [5]. Renowned for various biological activities such as antimicrobial, antioxidant, anti-cancer, and wound-healing properties, *R. canescens DC* has been extensively studied [6]. The utilization of natural food additives, or “phytochemicals,” derived from plants has witnessed a surge in popularity [7]. However, the volatility and instability of many phytochemicals in the gastrointestinal tract or atmospheric conditions can result in a loss of functionality [8]. To address this issue, these compounds are often encapsulated in phytosomes [9] or liposomes, small edible particles that shield bioactive compounds from atmospheric oxygen while facilitating a gradual release for extended functionality [10]. The liposome nanoformulation technique seeks to generate monodisperse particles, attain the appropriate lamellarity, incorporate drugs efficiently, and provide prolonged colloidal stability [11].

Across human history, medicinal plants have been harnessed for their functional attributes. Serving as primary or secondary metabolites, plants exhibit the capacity to produce and store a wide range of compounds with diverse structures and bioactivities [12]. Within these compounds, phenolic volatile aromatic substances have been a focal point of numerous studies, revealing their dual antimicrobial and antioxidant properties. This underscores the potential for formulating these natural compounds into agents with antimicrobial and antioxidant capabilities, not only applicable in the food industry but also in various other domains [13]. Coumarin and its derivatives, such as 1,2-benzopyrone or 2H-1-benzopyran-2-one, are naturally occurring compounds present in plants either as heterosides or in a free form. These compounds have gained prominence in recent years owing to their diverse biological activities, encompassing antibacterial, antifungal, and anticoagulant properties. Additionally, they exhibit a robust antioxidant and anti-oxidative stress effect by scavenging reactive oxygen species [14].

Coumarins are present in both the industrial and food additive sectors, but their most prevalent application is within the pharmaceutical industry. Here, they play a pivotal role in the synthesis of various synthetic pharmaceutical products [15]. Soft white cheese (SWC) faces challenges with a limited shelf life, even under refrigeration, owing to the presence of diverse contaminating microorganisms that can endure standard storage conditions during manufacturing and handling. These microorganisms include *Staphylococcus aureus, Escherichia coli, Listeria monocytogenes*, and *Salmonella typhimurium* [16]. SWC is susceptible to microbial spoilage and pathogenicity due to its brief shelf life, with a risk of oxidation reactions during processing and consumption, especially upon opening the package. Typically, no additional processing is applied to maintain the freshness of SWC, which can result in spoilage. Surprisingly, there is a lack of research on the potential use of nanoliposomes containing coumarin to enhance the microbial quality of SWC. In our previous research, it was identified that among various crude extracts tested, *R. canescens* DC CE demonstrated the highest levels of activity when screening for antioxidant and antibacterial properties [17].

Therefore, this study aimed to identify the functional constituents in *R. canescens* DC CE using liquid chromatography-mass spectrometry (LC-MS), investigate the antioxidant properties of coumarin and its antibacterial properties in terms of the minimum inhibitory concentration (MIC) and the minimum bactericidal concentration (MBC) against *P. aeruginosa*, *E. coli*, *Staph. aureus*, *S. typhimurium*, and *L. monocytogenes*, formulate and characterize physically stable liposomes incorporating coumarin, optimize their encapsulation efficiency (EE), and investigate their antibacterial activity. The antibacterial activity was assessed in vacuum-packaged SWC inoculated with *S. aureus, S. typhimurium, and P. aeruginosa.*

## Materials and methods

### Analytical LC-MS analysis

LC-MS is a highly sensitive and specific analytical technique. The combination of liquid chromatography (LC) and mass spectrometry (MS) is referred to as LC-MS. Component separation can be accomplished using LC, and the LC sample eluents are then transferred to MS, where detection, identification, and determination of component masses in the presence of other components can be accomplished [18]. At an environmental laboratory (Amman, Jordan), the components of *R. canescens* DC CE were determined using LC-MS (X500R QTOF system 2017, AB Sciex Pte. Ltd., Singapore). The MS device attached to the experiment was able to detect the CE that was eluted after conducting LC separation. The separation was performed on a Kinetex column (Phenomenex, Germany). measuring 100 × 2.1 mm and packed with C18 material of 1.7 μm particle size. A flow rate of 0.3 mL/min and a column temperature of 40ºC were used during the experiment. An injection volume of 10 µ L was used, and the data was collected using the Chromeleon® software connected to the instrument. The mobile phase used during the experiment consisted of a mixture of 65% methanol and 35% acetonitrile. The chemical compounds in the extract were identified by matching their spectra to those in the NIST Libraries, which were delivered by the instrument software. To quantify *R. canescens* DC CE, LC-MS used a peak area ratio of the selected ion collected (SIM) signal of the analyte and the internal standard, as described [19].

### Antioxidant activity of coumarin (free radical scavenging activity)

The free radical scavenging activity of coumarin was evaluated using the 2, 2-Diphenyl-1-picrylhydrazyl (DPPH) assay. When reduced by an antioxidant, the DPPH radicals can be detected by absorption at 517 nm. The higher the reduction in absorption capacity, the higher the antioxidant capacity. In brief, a 0.1 mM DPPH solution in methanol was prepared, and 4 mL of this solution was separately added to 1 mL of coumarin or ascorbic acid in methanol as a positive control. After 30 minutes of incubation at room temperature, the absorbance at 517 nm was measured using a UV-visible spectrophotometer (Biotek, MO, USA). The lower the absorbance of the reaction mixture, the higher the free radical scavenging activity. The ability to scavenge the DPPH radical was calculated using the following equation:


$$\text{\% DPPH Scavenging Effect}=\frac{\text{A control}-\text{A sample}}{\text{A sample}}\text{x }100$$ (1)

Where A is the absorbance measured at 517nm.

### MIC and MBC of coumarin against different bacterial strains

MIC values were calculated using the 96-well micro-dilution method [20]. 100μL of overnight culture containing 10^5^ CFU/mL of each bacterium was added to each well. Coumarin 100 mg/ml of dimethyl sulfoxide (DMSO) were subjected to serial two-fold dilution. Each 80 μL of the bacterial suspensions was added to 20 μL of each serial two-fold dilution of the test material in a 96-well dish plate and then was mixed [21]. The plates were then closed and incubated for 24 hours at 37^°^C. A microplate reader was used to measure absorbance at 600 nm (ELX 800, Biotek, Highland Park, VT, USA). Negative controls included 0.05% DMSO, uninoculated M-MRS broth, and water were also added to separated wells. MIC was determined as the lowest concentration of coumarin that had a significant difference in absorbance from the controls. MBC was determined using the pour plate technique (Muller Hinton Agar) [22], on which 20μL aliquots from clear wells were plated. When no visible growth was observed on the agar plates, MBC was believed to have the lowest concentration of coumarin.

### Preparation of coumarin liposomes

#### Qualitative and quantitative analysis of coumarin.

To ensure the identity of the purchased standard coumarin (Sigma-Aldrich), its chromatogram was compared to the pure coumarin chromatogram based on the method described as follows: Standard coumarin (conc. 5 mg/mL) was dissolved in ethanol and injected into a DIONEX UltiMateTM3000 High-Performance Liquid Chromatography HPLC (Thermo Fisher Scientific, Waltham, MA, USA). Coumarin was detected using a UV-VIS-PDA Detector at 274 nm, a Kromasil® C18 column (150 mm x 4.6 mm, 5m), a flow rate of 1.0 mL/min, a column temperature of 30^o^C with a 20 µ L injection volume, and computer software Chromeleon®. The mobile phase ratio was 40:60 acetonitrile. Retention times were compared to qualitatively detect the presence of standard coumarin [23].

#### Liposomes formation by ethanol injection method.

The ethanol injection method is based on using an organic solvent (ethanol) in which lipids (phosphatidylcholine (PC)) and the hydrophobic active agents (coumarin) are quickly injected into an aqueous phosphate buffer [24, 25]. Utilizing ethanol for injection required a 10-to-20-fold volume aqueous phosphate buffer than that of the injected ethanol solution and ethanol to be evaporated later on under vacuum using a rotary evaporator, dialysis, or filtering. This method mostly prepared liposomal formulations with higher polydispersity indexes (PDI) [26]. In addition, continuous exposure to high temperatures and organic solvents might reduce drug and lipid stability, which could be reduced by using ethanol as its low evaporation temperature [25].

The ethanol injection method was used to form liposomes consisting of a PC monolayer containing coumarin and an aqueous phosphate buffer saline (PBS) solution to be injected with an ethanol solution containing PC and coumarin. Different liposome formulations containing coumarin: PC in different weights (100:100, 50:100, 100:10, 10:100, and 100:50 (mg/mg), respectively) were dissolved in 1 mL of ethanol. Each of the resulting organic mixtures was separately vortexed for two minutes and sonicated for 5 minutes at 35^°^C using a bath sonicator, then injected in 9 mL of 0.01 M PBS solution (pH 7.4) containing 137 mM NaCl, 2.7 mM KCl, 8 mM Na_2_HPO_4_, and 2 mM KH_2_PO_4_ placed on a hot plate with magnetic stirring. When the ethanolic solution came into contact with the aqueous phase, it spontaneously formed liposomes. The liposome suspension was then stirred at room temperature for 1-2 hours. Finally, using a hot plate with magnetic stirring, the ethanol and some of the PBS were removed. After the formulation of the nanoliposomes, EE, drug loading (DL), and stability were tested for different formulations. First, to ensure the stability of the liposomes and their ability to encapsulate the coumarin, the liposome-coumarin solution was dialyzed using a dialysis membrane and soaked into PBS overnight. Then the loaded coumarin concentration was measured for liposomes before and after dialysis by rupturing the liposomes using acetonitrile and measuring the released coumarin [27, 28]. The prepared liposomes used for characterization and other tests were used as colloidal dispersion after preparation.

### Characterization of coumarin nanoliposomes

#### Shape and Surface Morphology.

A Transmission Electron Microscope (TEM) (Versa 3D. FEI Company, Netherlands) was used to determine the shape and surface morphology (roundness, smoothness, and aggregate formation) of the nanoliposomes, as well as their size distribution. A drop of liposome suspension was placed in a glass slide. The drop content was heat dried and fixed, then viewed and photographed the surface morphology using the TEM.

#### Hydrodynamic diameter and zeta potential.

The average hydrodynamic diameter, zeta potential, and PDI of free liposomes in the suspension were determined using Dynamic Light Scattering (DLS) on a Zetasizer (Malvern Instruments Ltd., Malvern, UK). The samples were diluted 1:20 with distilled water before the analysis (attenuator reading of 7).

#### Encapsulation efficiency (EE).

Liposomes were disrupted by adding 400μL of HPLC-grade acetonitrile to 100μL of liposomes, then sonicating for 5 minutes at 55 ^°^C and centrifuging for 10 minutes at 12000 rpm. The resulting mixture was then filtrated through a 0.45μm syringe filter before being injected into the HPLC [29]. The EE and DL were calculated as follows:


$$\text{EE }\left(\text{\%}\right)=\frac{\text{Entrapped coumarin}}{\text{Total coumarin}}\times 100$$ (2)

$$\text{DL }\left(\text{\%}\right)=\frac{\text{weight of loaded coumarin}}{\text{weight of lipids}}\times 100$$ (3)

### The food model (SWC)

#### Manufacturing of SWC.

The SWC was manufactured in the dairy factory of the Faculty of Agriculture, University of Jordan. Pasteurized cow milk (10 kg, 37^°^C) at 45^°^C was coagulated within 40-60 minutes of adding rennet (1.7 g) and 0.1 g of calcium per 10 L of milk. The produced coagulant was pressed through cheesecloth. Following that, the cheese was vacuum-packed and stored at 4^°^C for 30 days [30].

#### Antibacterial activity of coumarin liposome and coumarin alone against bacteria strains in the SWC.

*P. aeruginosa, S. typhimurium, and Staph. aureus* (approximately 105 CFU/mL of each) were inoculated into SWC samples before the final step of shaping and pressing. The inoculated cheese curd was then kept at room temperature for 30 minutes under aseptic conditions to allow bacterial attachment and adaptation. Before adding the treatments (coumarin liposome and coumarin alone) to the SWC, the same concentration (10 mg/10 mL) of both liposome and coumarin alone was sterilized under aseptic conditions (laminar flow) using a 0.45µm filter at the CTC laboratory. Coumarin liposome formulation (10:100) was chosen to be added to SWC based on the best characterization of different ratios of PC to coumarin compared with other liposome formulations. Coumarin liposome and coumarin alone were added to the inoculated SWC samples at the level of 1 mL/100 g of cheese [31]. All samples were placed in sterile bags and vacuum packaged, then stored at 4 ^°^C. As a control, an uninoculated cheese sample with no treatments was also prepared in the same manner. All samples were repeated twice at 0, 7, 14, 21, and 30 days.

### Statistical analysis

Data was analyzed to determine significant differences between groups using one-way analysis of variance (ANOVA) followed by Dunnett’s post hoc test. The results from 3-4 independent experiments were presented as means ±  standard deviation (SD). To determine the statistical differences between the control and treatment groups, a GraphPad Prism ANOVA followed by Dunnett’s post hoc test was used. A p-value ≤  0.05 was considered statistically significant for all analyses. Highly significant statistical differences were indicated by p values of ≤  0.001.

## Results

### LC-MS analysis of chemical composition of *R. canescens* DC CE

**Fig 1:**
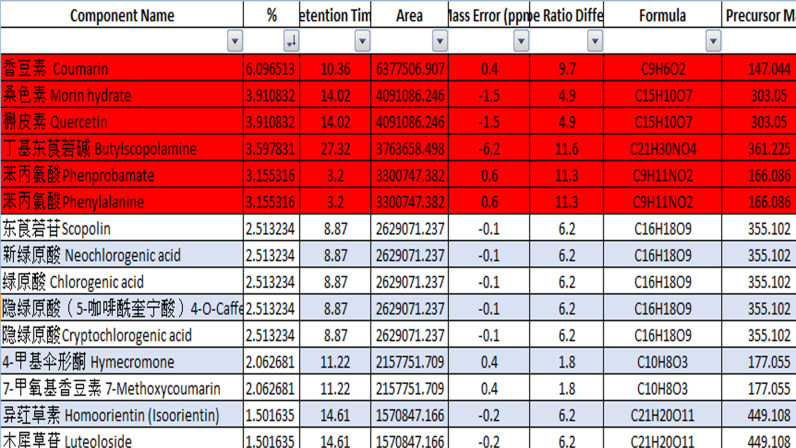
Major components of *R. canescens* DC CE screened by LC-MS.

**Fig 2:**
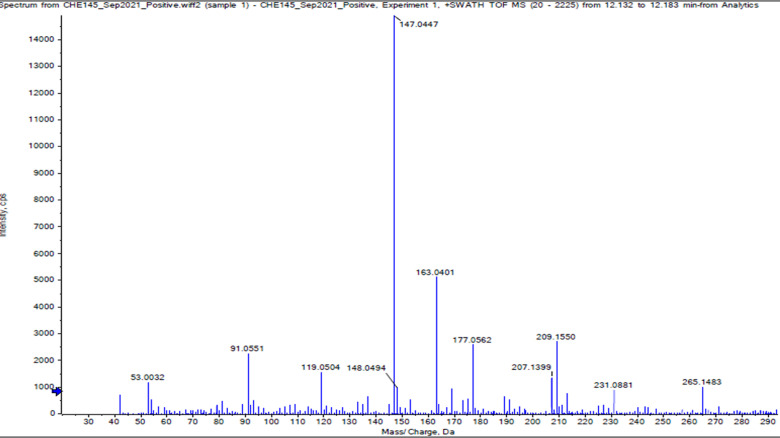
LC-MS chromatogram for natural product screening of *R. canescens* DC CE shows coumarin has the highest peak between different flavonoid compounds.

Fig 1 and Table 1 show the major components of *R. canescens* DC CE screened by LC-MS with their % presentation in relation to other compounds in the CE. Fig 2 represents the LC-MS chromatogram that showed the relative peak height and area of the *R. canescens* DC components. The main components of *R. canescens* DC CE were coumarin, then morin hydrate, followed by quercetin, butylscopolamine, and phenylalanine. The other components are presented in lower and minor percentages. Similar results were found by Jirovetz *et al.* [32], who found that flavonoid compounds such as coumarin are the major identified compounds in the *R. canescens* DC CE [32].

**Table 1:** Major natural components of *R. canescens* DC CE and their product screening (%) of CE.

Compounds	Percentage presentation to total compounds (%)
Coumarin	6.09
Morin hydrate	3.91
Quercetin	3.91
Butylscopolamine	3.59
Phenylalanine	3.15

Several other studies have revealed that the main components of *R. canescens* DC extract are coumarin, morin hydrate, quercetin, butylscopolamine, phenprobamate, and phenylalanine, which are similar to our results (Fig 2 and Table 1). Therefore, in the current study, potential antioxidant and antibacterial activities of the coumarin were tested, as it was found to be the major component of *R. canescens* DC CE. To do so, a purified form of coumarin was purchased from a supplier; its identity was confirmed before it was further tested for functional properties.

### Antioxidant activity of coumarin

**Fig 3:**
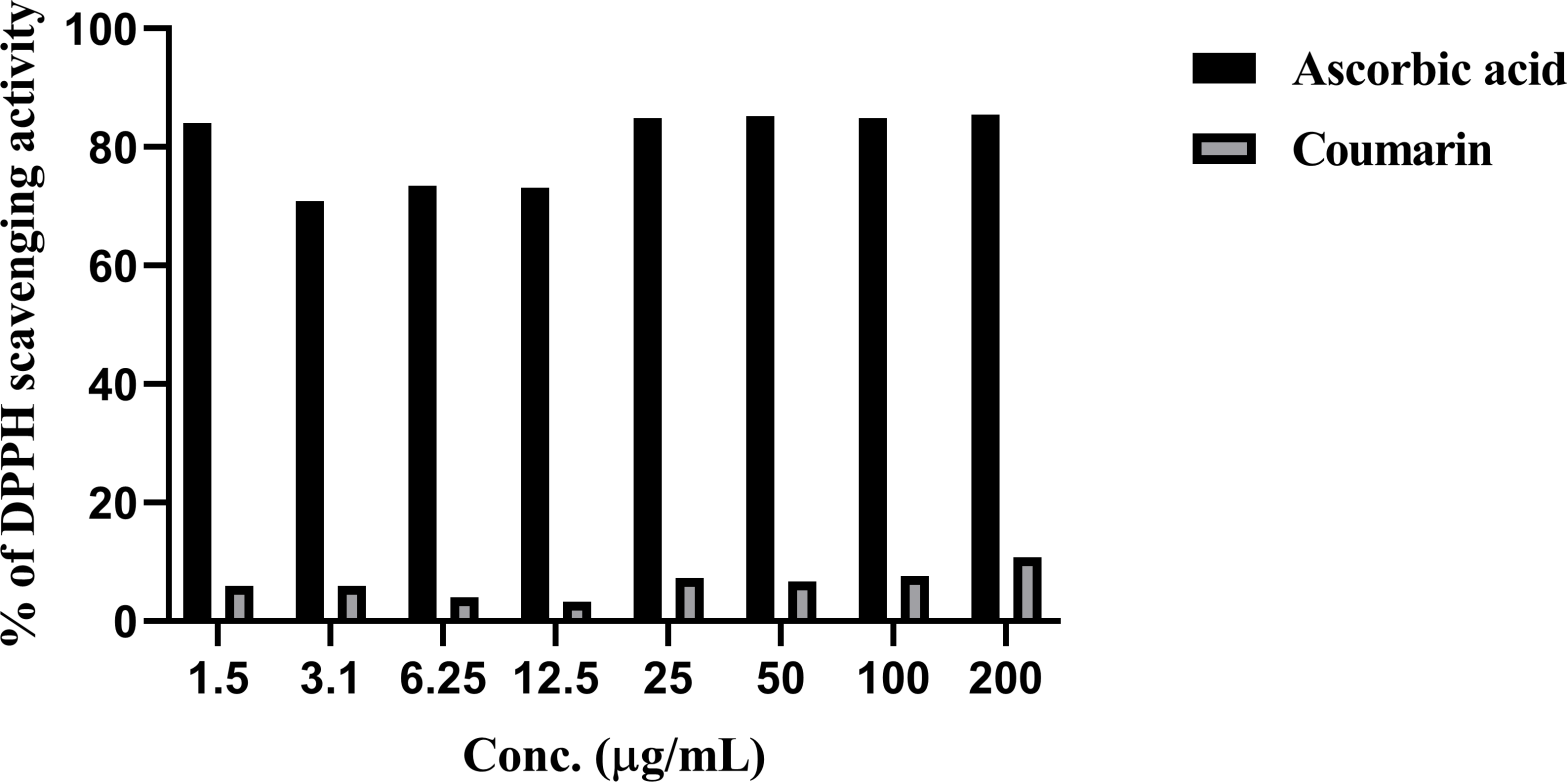
Antioxidant activity of coumarin and ascorbic acid. The results represent percentage of DPPH scavenging activity. Ascorbic acid was used as positive control at 100 mg/ml. Results are expressed as means ±  SD (n =  3 independent replicates).

Fig 3 shows the antioxidant activity of coumarin and ascorbic acid. The DPPH scavenging effect was used to evaluate the antioxidant activity of coumarin and ascorbic acid. The results show that, when compared to the ascorbic acid scavenging effect, coumarin had an insignificant effect p ≥ 0.05.

### Antibacterial activity of coumarin

The antibacterial activity of coumarin was evaluated in terms of MIC and MBC. Based on the results in Table 2, the MIC and MBC values for coumarin were 2.5 and 5 µg/ml against *P. aeruginosa*, 2.5 and 5 µg/ml against *E. coli*, 2.5 and 5 µg/ml against *Staph. aureus*, 1.25 and 2.5 µg/ml against *S. typhimurium*, and 1.25 and 2.5 µg/ml against *L. monocytogenes*, respectively.

**Table 2:** MIC and MBC values (µg/mL) of coumarin against different bacterial strains.

Bacterial Strains	MIC (µg/ml)	MBC (µg/ml)
*P. aeruginosa*	2.5	5
*E. coli*	2.5	5
*Staph. Aureus*	2.5	5
*L. monocytogenes*	1.25	2.5
*S. typhimurium*	1.25	2.5

### Qualitative analysis of coumarin

**Fig 4:**
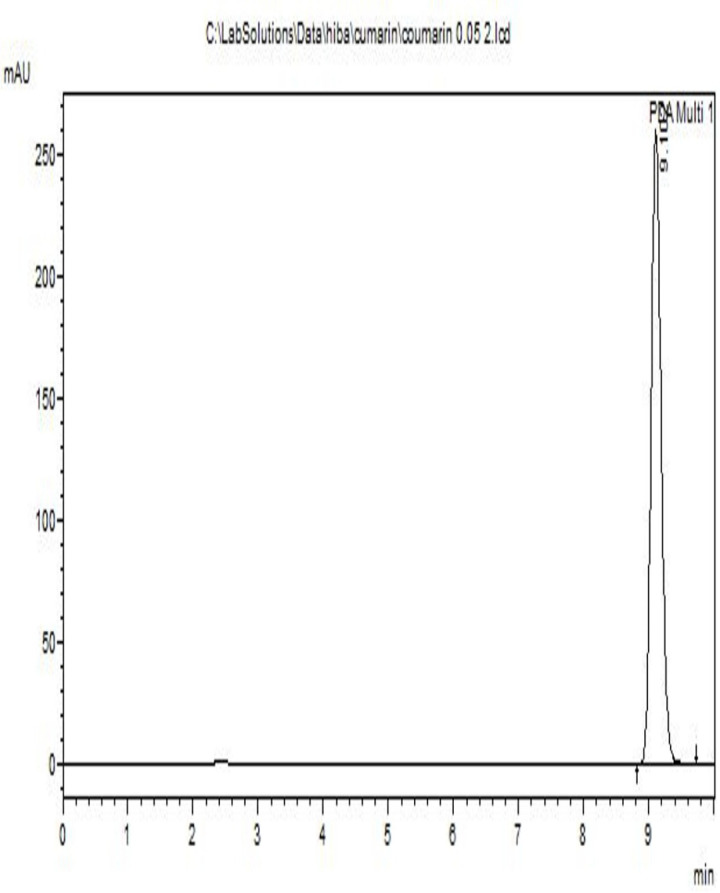
HPLC chromatogram for the standard coumarin.

Fig 4 shows the HPLC chromatogram for qualitative analysis of pure coumarin (99% HPLC) and its retention time. The compound was identified by its retention time based on the previous findings that were done under the same experimental conditions [23,33,34]. The chromatogram shows that a sharp peak was detected at 9.1 minutes. No other impurities were detected together with coumarin. Other research showed that the peak appeared at 9.0 minutes under the same experimental conditions, indicating that the identified compound was coumarin [23].

### Characterization of coumarin nanoliposomes

#### Shape and surface morphology.

**Fig 5:**
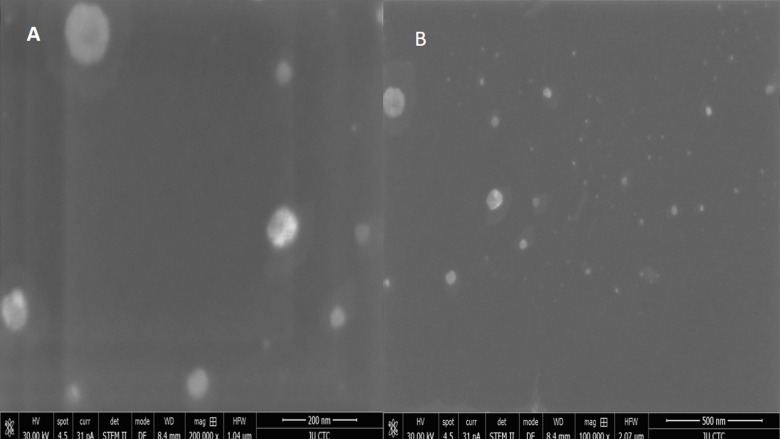
TEM micrographs of coumarin liposomes at different magnifications. (A) at 200nm. (B) at 500nm as determined by the TEM.

The shape of the prepared nanoliposomes (PC: coumarin (10: 100)) was regular spherical with various sizes and their surfaces were rough as shown by the TEM micrograph (Fig 5).

#### Hydrodynamic diameter and zeta potential.

**Fig 6:**
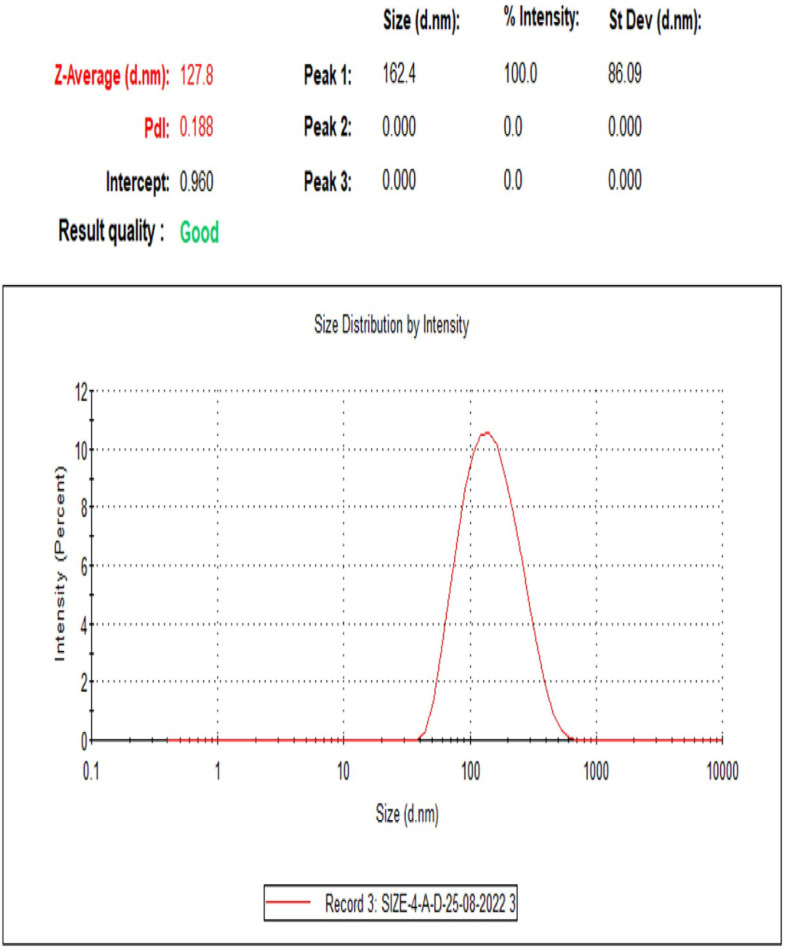
Particle size distribution and polydispersity index of nanoliposome dispersion [100:10; PC: coumarin] obtained by DLS; showing single peak (unidistribution).

Hydrodynamic diameter, PDI, and zeta potential are all critical parameters in the field of nanoparticles. The mean ±  standard deviation of these variables prepared with different formulations of the liposomes are shown in Table 3, whereas the size distribution by DLS graph is shown in Fig 6.

**Table 3:** Hydrodynamic diameter, PDI and zeta potential of coumarin liposome as mean ± SD.

Liposome formula (PC: coumarin)	Hydrodynamic diameter	PDI	Zeta potential
**10:100**	73.7 ± 6.8	0.73 ± 0.05	-65.3 ± 3.6
**50:100**	173.2 ± 4.04	0.19 ± 0.05	-60.4 ± 4.04
**100:100**	170 ± 1.84	0.16 ± 0.06	-59.3 ± 2.4
**100:50**	140.8 ± 3.7	0.21 ± 0.03	-58.6 ± 2.7
**100:10**	127.8 ± 0.3	0.36 ± 0.35	-61.03 ± 2.9

### Encapsulation efficiency (EE)

**Fig 7:**
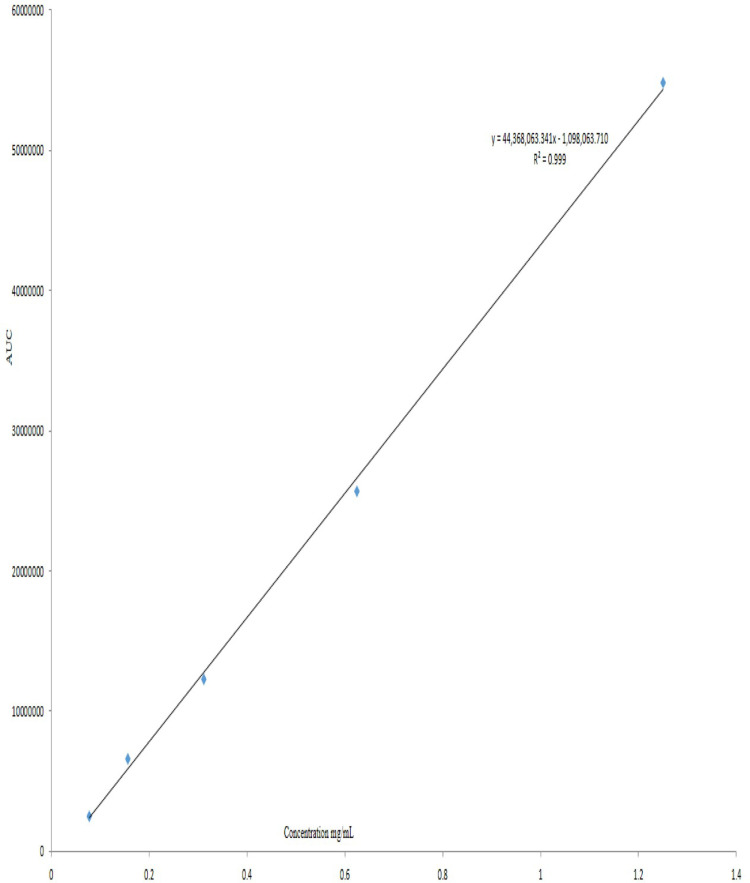
Calibration curve of coumarin concentration vs. area under curve using HPLC.

Fig 7 shows the linear calibration curve of coumarin concentration vs. area under curve. The curve shows a very high correlation coefficient (R^2^ =  0.999), which was used to calculate EE and DL.

**Fig 8:**
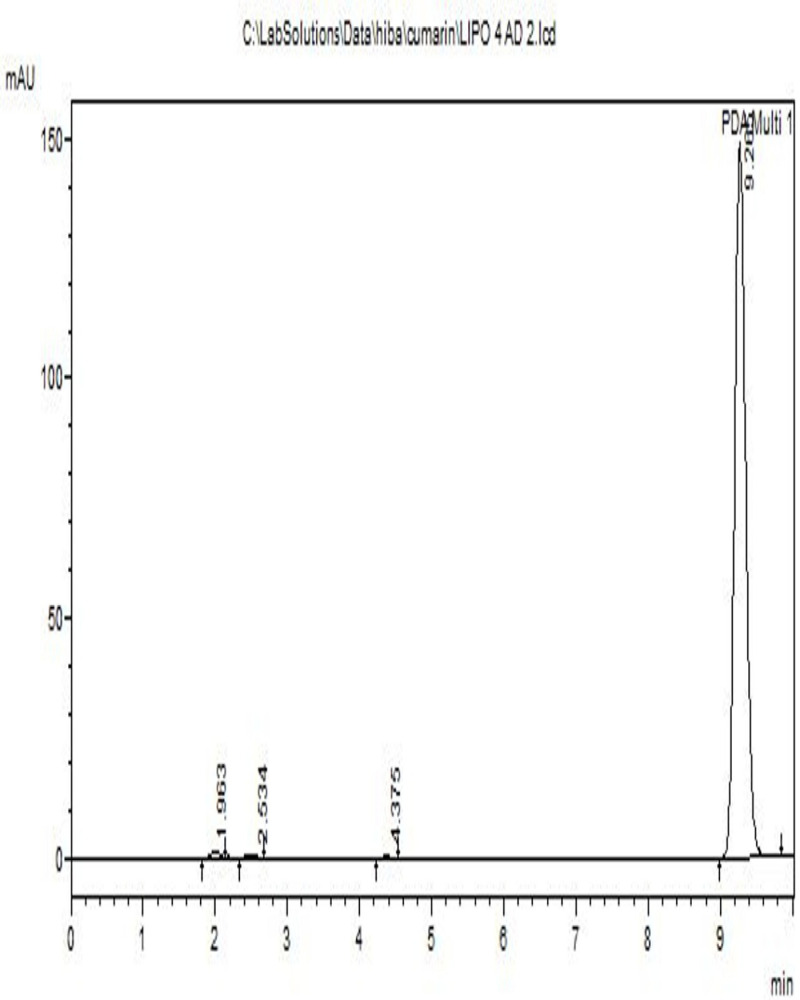
HPLC chromatogram of coumarin entrapped inside the liposomes.

EE represents the total amount of pure coumarin entrapped in liposomes in relation to the amount before entrapment. Fig 8 shows a representative chromatogram of the coumarin measured at 274 nm after the liposome’s destruction in the EE study. The retention time of the detection was 9.25 min, indicating that this peak represents the coumarin. EE and DL were calculated for different liposomal preparations of PC and coumarin (10:100, 50:100, 100:100, 100:50, and 100:10), respectively; the means ±  standard deviation of these variables prepared with different formulations of the liposomes are shown in Table 4, which indicates that liposomes formulated are in the nanoscale.

**Table 4:** EE and DL of coumarin liposome.

Liposome formula (PC: Coumarin)	EE (%)	DL (%)
**10:100**	1.39 ± 0.4	13.87 ± 0.42
**50:100**	4.1 ± 0.5	8.2 ± 0.51
**100:100**	4.15 ± 0.3	4.15 ± 0.3
**100:50**	2.75 ± 0.1	1.38 ± 0.5
**100:10**	40.93 ± 0.2	0.05 ± 0.001

### Antibacterial activity of coumarin against bacterial strains inoculated in SWC

**Fig 9:**
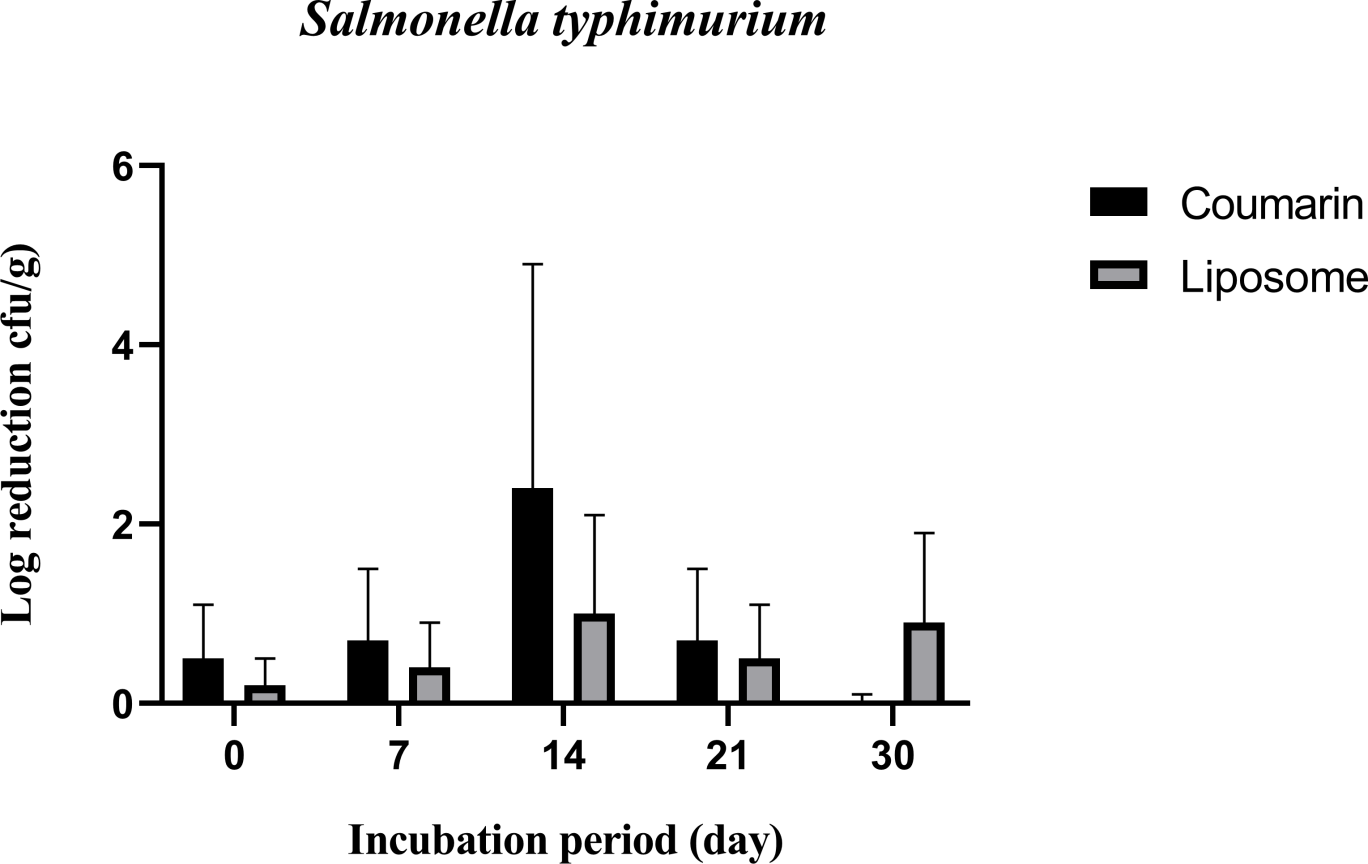
Antibacterial activity of coumarin liposome and coumarin against *S. typhimurium* inoculated in SWC during storage at 4˚C for 30 days. All experiments were conducted in triplicates.

Fig 9 shows that coumarin liposome and coumarin alone treatments have antibacterial activity against *S. typhimurium* in SWC stored at 4˚C for 30 days. When coumarin alone was added to the cheese, the *S. typhimurium* decreased in SWC by 2.4 log CFU/g compared to the control at 14 days of storage, then the numbers increased rapidly at 21 days of storage (with reductions of 0.4 and 0.2 for coumarin and liposomes, respectively). The coumarin liposome kept a lower and more steady number with counts kept between 0.9 and 0.1 log CFU/g, inducing it’s the inhibitory effect. Finally, it has been found that both coumarin alone and coumarin liposomes are equally effective in inhibiting the growth of *S. typhimurium* populations. Significant differences (P ≤ 0.05) in log reduction of coumarin liposome only were detected at 30 days storage.

**Fig 10:**
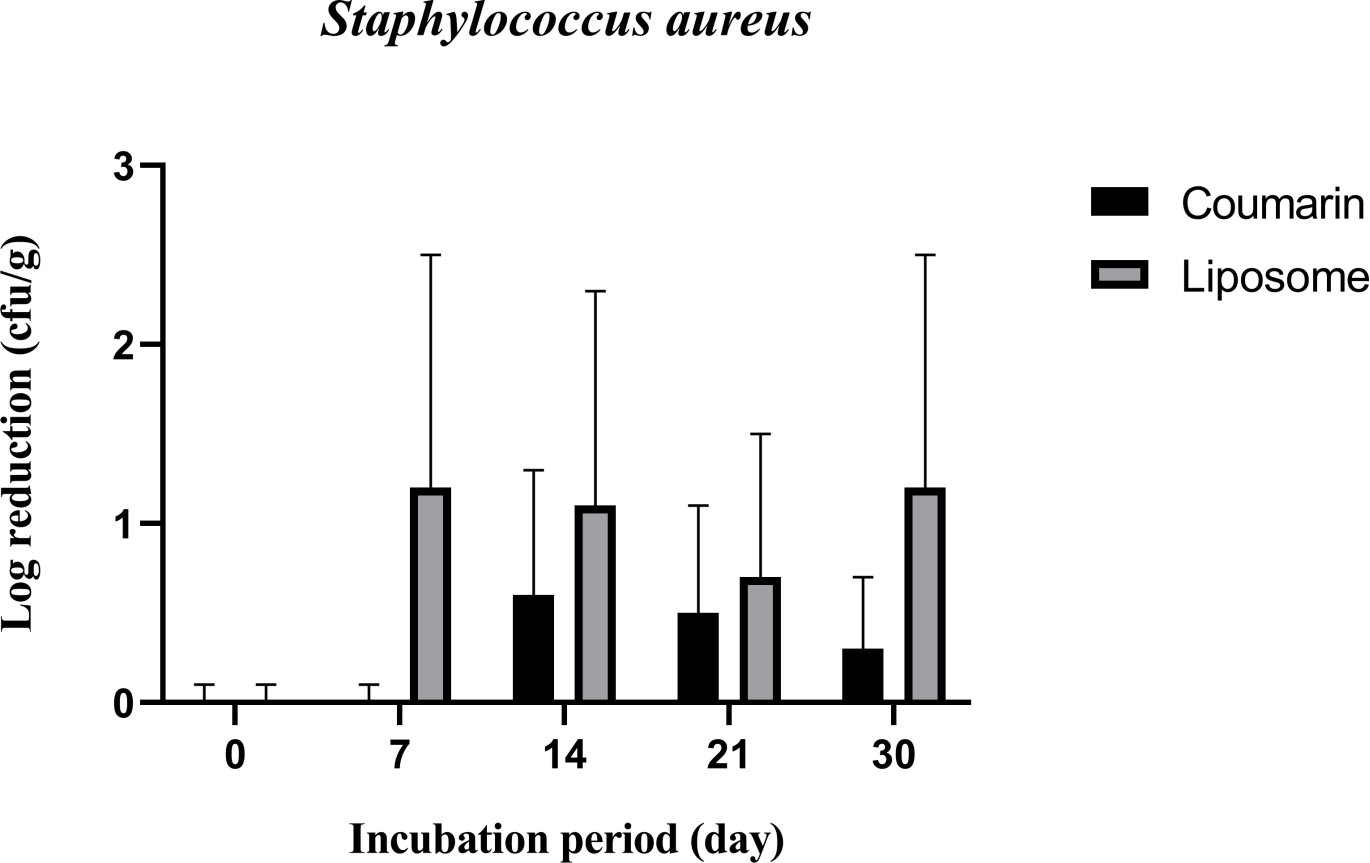
Antibacterial activity of coumarin liposome and coumarin against *Staph. aureus* inoculated in SWC during storage at 4˚C for 30 days. All experiments were conducted in triplicates.

Fig 10 shows that both coumarin liposome and coumarin alone have more inhibitory effect against *Staph. aureus* than *S. typhimurium* in SWC during storage at 4˚C. Again, more inhibitory against Gram-positive than Gram-negative bacteria. Both types of treatments have about the same antibacterial activity against *Staph. aureus,* and there were no significant differences in their effect during the storage period. The log reduction of *Staph. aureus* in SWC treated with coumarin liposome increased to 1.2 log CFU/g at 7 days of storage, then it stayed constant throughout the storage period of 30 days. While those treated with coumarin alone had lower but not significant log reductions. Significant differences (P ≤ 0.05) in log reduction of coumarin liposome only were detected at 30 days storage.

**Fig 11:**
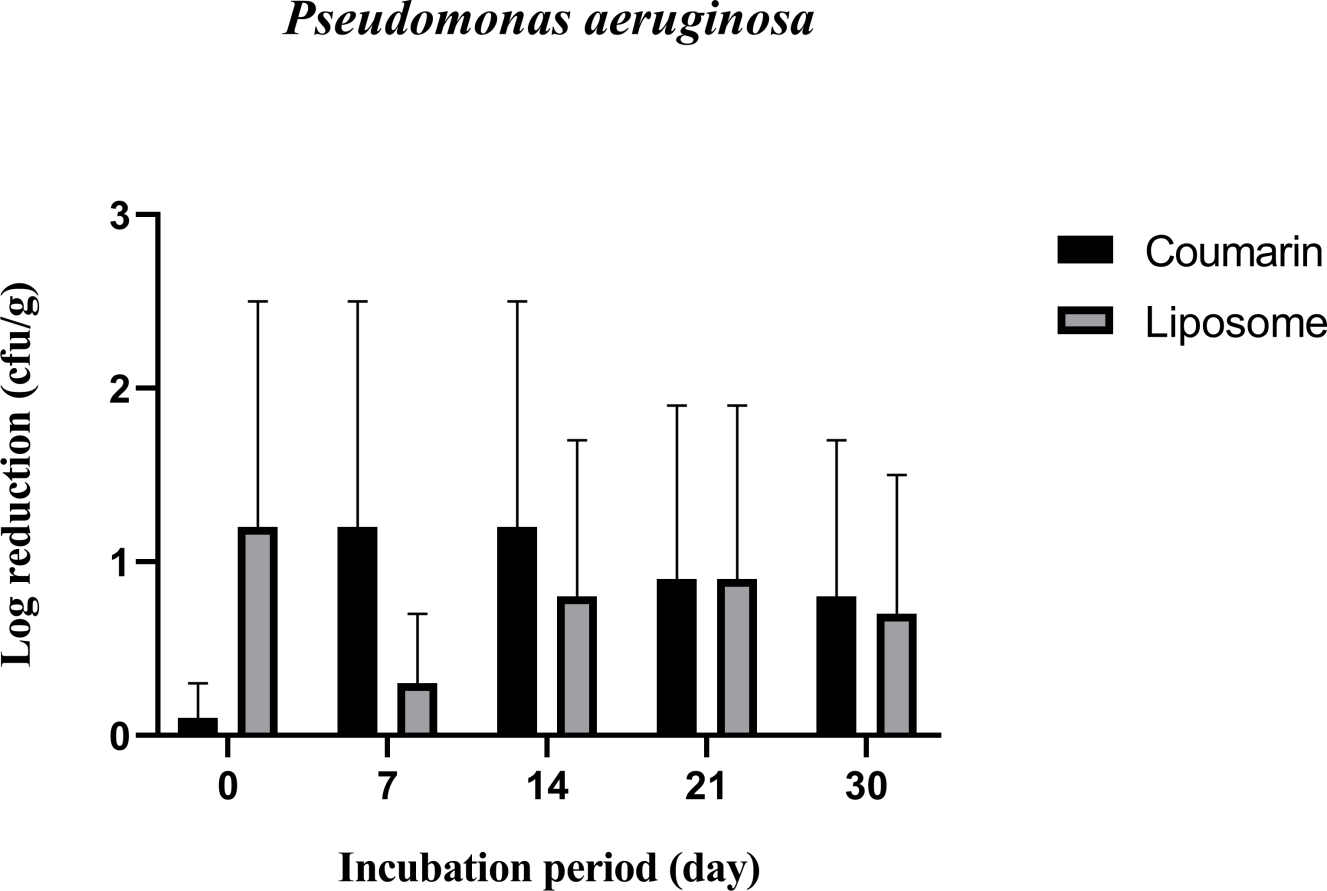
Antibacterial activity of coumarin liposome and coumarin against *P. aeruginosa* inoculated in SWC during storage at 4˚C for 30 days. All experiments were conducted in triplicates.

Fig 11 shows that coumarin liposome and coumarin alone treatments have similar inhibitory effects against *P. aeruginosa* count in SWC during storage at 4˚C for 30 days. Both treatments appeared to be inhibitory against *P. aeruginosa* and *Staph aureus* (Fig 10) more than *S. typhimurium* (Fig 9), with log reductions ranging between and for coumarin alone and between and for liposomes throughout the storage period (Fig 11). It was also observed that, after 7 days of storage, both coumarin liposome and coumarin alone had similar inhibitory effects on the growth of *P. aeruginosa* in SWC during storage at 4 ^°^C.

## Discussion

Although coumarin was the major component of the *R. canescens* DC CE (Fig 1 and Table 1), it has low free radical scavenging activity (Fig 3). Whereas *R. canescens* DC had the highest total phenol content, free radical scavenging activity, and reducing power compared to other plants as in our previous study [17]. This could indicate the antioxidant activity could be due to the mixed components in the *R. canescens* DC rather than one component. Similar results were found by Assafiri *et al.* [35] when tested components of *the Rubus* ulmifolius plant [36]. However, the mechanism of DPPH scavenging activity is not fully understood, and the correlation of effects with chemical structure and concentration is inconclusive at this time [37]. In another study, it was reported that the antioxidant properties of coumarins could be determined by a variety of molecular action mechanisms that are likely related to their structural similarity to flavonoids and benzophenones [38]. Because coumarins differ in the degree of oxygenation due to their content of benzopyrane moiety, the synergistic effect of different phenolic compounds in *R. canescens* DC extract may be greater than that of coumarin alone [37]. In terms of antibacterial activity, *R. canescens* DC CE exhibited superior bacterial inhibition compared to coumarin, as shown by lower MIC and MBC values in our earlier work [17].

According to Liang and Chou [39], the liposomes demonstrated a spherical morphological structure with smooth surfaces, and they were uniform in size with a homogenous distribution [39]. There were no visible agglomerations of coumarin nanoliposomes in the carrier system. Most of the particles ranged between 73.7 to 173.2 nm and had a predominant spherical shape. The TEM images of the current study show structural variations in the 10: 100 (PC: coumarin) formula of the liposome, which suggested that the liposome nanoparticle structure was affected by the PC ratios [40].

The 10:100 formulation had the lowest hydrodynamic diameter, the highest PDI, and the highest negative z-potential, which means that this formulation has the highest stability as it has the lowest size, thus weight, light scattering, and the highest repulsion. It was reported that PDI is a measure of the heterogeneity of the particle size distribution, with a value of 0.0 indicating a monodisperse sample (all particles are the same size) and a value of 1.0 indicating a highly polydisperse sample (particles have a wide range of sizes). In general, a low PDI value indicates a more uniform distribution of particle sizes and is desirable for many applications, including in the fields of pharmaceuticals and nanotechnology [41]. The highest negative z-potential indicates the highest repulsion between particles (Table 3). It was reported that the amount of the encapsulation material (PC) to the encapsulated material (coumarin) dramatically affects liposome stability. This combination may affect particle size, which is associated with PDI. Also, it may affect the z-potential, thus the repulsive force between the particles that prevents aggregating and settling down of the particles [42]. It can be concluded that 100:10 was the best formulation and therefore was chosen for further experiments. Particle size distribution and PDI of nanoliposomes (Fig 6) showed that only a single peak was detected, which has a normal distribution (mean 162.4 nm). Three colored lines represent that the same zeta potential was taken three times. This is also confirmed by the high PDI obtained (Table 3).

Although the 100:10 formulation had the highest EE and DL, it had the lowest dispersibility and stability (Table 4). This could be related to the higher content of the heavier material PC (as it contained essential oils of the plant) that increase the weight of these liposomes and decrease their stability. One of the objectives of this study is to create a new liposomal carrier for coumarin, which is thought to have, if present, anti-microbial antioxidant activities. First, coumarin was qualitatively analyzed using HPLC in the *R. canescens* DC CE. This compound was loaded into PC liposomes that were prepared using an ethanol injection method. The prepared liposomes were characterized using a variety of techniques, including hydrodynamic diameter, PDI, and zeta potential, which were all calculated using DLS. For a particle population to be considered nanoparticles, 50% of the particles in the population should have at least one dimension in the range of 1-100 nm in the number size distribution [43, 44]. However, the size range of organic nanoparticles is slightly wider; for example, lipid-based nanoparticles can range from 10-1000 nm [41]. Using that definition, our liposomes are considered nanoparticles, as their average hydrodynamic diameter was 127.80.3 nm. Liposomes’ size and charge are important indicators for the liposome’s stability. Small particles (diameter 200 nm) have the desired physicochemical properties and bioavailability for long-term release of encapsulated active substances [31]. Particle size and size distribution are critical factors in determining dosage form stability during storage. According to our findings, coumarin has a suitable size range and can maintain stability [45]. PDI is a unitless number that represents the diversity and range of particle size distribution range. It can range from less than 0.05, indicating a monodispersed population, to 0.7, indicating a population with a very wide particle size distribution range [42]. The particle size of liposome nanoparticles is extremely important as it can affect both the stability and the bioavailability of crude extract. Smaller particles have a larger surface area and therefore have a faster release in addition to higher stability, allowing them to pass through the cell membrane fluently [40]. As a monodispersed population, a PDI of 0.3 is considered acceptable for liposomes [42]. As a result, our liposomes are classified as nanoparticles with PDI ranging from 0.16 ± 0.06 to 0.36 ± 0.05, indicating a relatively narrow particle size distribution [31].

Zeta potential is a measure of the stability of liposome dispersion against aggregation. It is defined as the electric potential difference between the stationary layer of liquid surrounding the particle and the dispersion medium, which is observed when the particle moves in response to an applied electric field [46]. Coumarin liposome surface zeta-potential values ranged from -65.3 ± 3.6 to -58.6 ± 2.7 mV. Historically, zeta-potential values between -30 and + 30 mV have been thought to indicate good stability, with no aggregation or precipitation [45]. As a result, the coumarin liposome can be considered a stable system. When measuring the zeta potential of nanoparticles, the strong negative surface charge revealed electrostatic repulsion between the particles. It is an important indicator of stability [45].

HPLC was used to determine the EE of coumarin liposomes. Coumarin standard curve was linear, with a very high correlation coefficient (R^2^ =  0.9999). The EE of coumarin in the liposome system ranged from 1.39 ± 0.4 to 40.93 ± 0.2%, according to the linear equation. The liposome EE reached the maximum value (40.93 ± 0.2%) for 100:10 formulation. This low value could be related to the reason that after the centrifugation step of EE measurement, a small proportion of the coumarin liposomes may have remained in the supernatant, resulting in coumarin loss. Furthermore, the type of lipids used has a significant impact on the EE [47]. The highest EE of our liposome is 40.93 ± 0.2%, which is considered high EE when compared to other liposomes [29].

Based on the above results, it was decided to engineer liposomes at a concentration of 10 mg/10 mL coumarin because it had the desired PDI, zeta potential, and EE. Although cholesterol is a common additive in liposomal formulations used to promote membrane rigidity, DL, and stability, for liposomes, a DL of 4–12% is considered acceptable as a stability population [42]. When compared to other liposomes, our liposome’s DL is 4.1 ±  0.3%, which is considered high EE. However, our liposomes were formulated from phospholipid only because cholesterol increases the amount of space taken up in the bilayer, leaving less room for drugs to pack into. This increased competition for packing space between cholesterol and drugs reduces the overall DL of the resulting liposomes and is also likely to increase the hydrophobicity of the liposome bilayer, implying that aqueous encapsulated in the liposome core is less likely to leak out [24].

It was reported that the mechanism of action of coumarin against bacterial cells, including *P. aeruginosa*, *S. typhimurium*, and *Staph. aureus,* is damaging the integrity of the cell membrane, which results in the loss of intracellular constituents and eventual cell death [31]. Because of its ease of availability and low cost, coumarin could be used in the food industry and agricultural applications. It has been determined that it is not genotoxic (not toxic to the DNA) and that it is safe for use in food products. Because of its effectiveness against both pathogens and not pathogenic bacteria such as *P. aeruginosa* and *Staph. aureus*, it was used to protect abiotic surfaces in food manufacturing or processing industries, poultry farms, vegetable farms, and meat industries [48]. Coumarins have previously been shown to have antibacterial activity against a wide variety of microorganisms. It was reported that based on coumarin structure, it is unlikely that coumarin had antibacterial activity by affecting the bacterial cells themselves, and rather than limiting microbial growth, it can target a key microbial cell-cell communication system and, with it, the ability to inhibit antibiotic-tolerant colonization structures known as biofilm [49]. Coumarin alone or coumarin liposome had the lowest antibacterial activity against *S. typhimurium* than the other microbial cells tested. Previous research found that strains of the *Salmonella* genus are resistant to plant extract; this could be explained by the widespread use of antibiotics against this group of bacteria [50]. However, our findings are consistent with previous research using pure molecules of flavonoids (coumarin). Unlike our results, these molecules alone outperformed CE in terms of antibacterial activity [51]. It was reported that coumarin has an effect on the early growth stages of *S. typhimurium* and their initial count, but this effect diminishes shortly after the treatment; similar results were found in the current study [48]. Results of the current study showed that *Staph. aureus* is sensitive to coumarin and thus was reduced by about 1 log reduction. It was suggested that coumarin treatment may impair *Staph. aureus* activity and growth by reducing the viable bacterial cells and/or weakening its ability to adhere to surfaces [52]. Because of its ability to inhibit the early stages of *Staph. aureus* growth (up to 7 days), our findings suggest that coumarin may be a promising compound to prolong the shelf life against *Staph. aureus-infected* foods.

Liposomes could control the release of coumarin, thus prolonging the inhibitory effect on the coumarin alone (Fig 10). The log reduction of the liposome-treated bacterial strains was nearly constant (approximately 1 log CFU/g). This may indicate that the prepared liposomes not only have good dispensability and stability but also contain a sufficient number of antibacterial agents that were released in a controlled manner over the storage period [31]. When compared to coumarin alone, coumarin liposome had a delayed but prolonged antibacterial effect; otherwise, compared to coumarin alone, coumarin liposome did not affect *Staph. aureus* [45].

The counts as count colony-forming units and assessment of the antibacterial efficacy exhibited a strong positive correlation. This indicates that a concentration of 10 mg of coumarin is efficient to control growth and multiplication of the tested bacterial strains. Liposome-encapsulated coumarin’s antibacterial activities and active time were significantly increased against *S. typhimurium for* 30 days when compared to coumarin alone. The improved stability of the coumarin by the developed liposome encapsulation and the higher functionality of such liposomes could be considered an innovation [31].

The results of the study suggest that urgent developments are needed to improve the quality and safety of milk and cheese products in Jordan [53]. The consumption of raw milk and fresh cheese in Jordan has been identified as a significant epidemiological factor in the spread of brucellosis [14]. To address this issue, the use of clean milk that is free of pathogens and has a low microbial load is recommended. Furthermore, the mandatory pasteurization of milk during cheese production, as required by the Jordanian standard for soft cheese, is crucial in ensuring the safety of the product. The implementation of good manufacturing practices during production is necessary to avoid cross-contamination, and the cooling chain must be maintained from milking to the consumption of the cheese. Additionally, the testing of soft white cheese for phosphatase should be included in governmental quality control testing of the product to ensure its quality and safety. The successful implementation of these measures requires collaboration between private and governmental stakeholders. In conclusion, the urgent need for improvements in the quality and safety of milk and cheese products in Jordan calls for the use of clean milk, mandatory pasteurization, good manufacturing practices implementation, maintenance of the cooling chain, and phosphatase testing. Private and governmental interested parties need to take action to ensure the successful implementation of these conditions [16].

## Conclusions

Our prior research identified *R. canescens* DC crude extract as exhibiting the highest antioxidant and antibacterial activity [17]. Coumarin, the main component of *R. canescens* DC, has notable antimicrobial and antioxidant properties. We developed highly stable, small-sized liposomes containing coumarin, which effectively reduced the growth of both Gram-positive and Gram-negative bacteria during refrigerated storage. These findings suggest potential applications in the food industry for enhancing food preservation and safety. However, further studies are needed to evaluate the safety, effectiveness, optimal concentration, application methods, and potential toxicological effects of coumarin-containing liposomes. Additionally, research should assess the stability of these liposomes under various storage conditions, their impact on the sensory qualities of food, and their shelf life compared to commercial preservatives. Exploring different lipids and encapsulation techniques could optimize the formulation and production process, potentially leading to new liposome-based delivery systems for applications such as drug delivery and gene therapy. Since *R. canescens* DC is edible and all nanoparticle preparation materials are food-grade, a food model incorporating these ingredients could serve as an anticancer and antimicrobial agent, warranting the transition from in vitro to in vivo model studies.

## Supporting information

S1 Raw data. Major Components of R. canescens DC CE. S2 Raw data. Major Natural Components of R. canescens DC CE. S3 Raw data. Liposome characterization. S4 Raw data. Antimicrobial Activity of Coumarin. S5. Raw data. Antioxidant Activity of Coumarin. S6. Raw data. Antioxidant Activity of Coumarin versus Ascorbic Acid. S7. Raw data. Encapsulation Efficiency of Coumarin.

## References

[1] Lourenço SC, Moldão-Martins M, Alves VD. Antioxidants of natural plant origins: from sources to food industry applications. Molecules. 2019;24(22):4132. doi: 10.3390/molecules24224132 31731614 PMC6891691

[2] Gilliland SE. Beneficial interrelationships between certain microorganisms and humans: Candidate microorganisms for use as dietary adjuncts 1,2. J Food Prot. 1979;42(2):164–7. doi: 10.4315/0362-028X-42.2.164 30812336

[3] Rashidi L, Khosravi-Darani K. The applications of nanotechnology in food industry. Crit Rev Food Sci Nutr. 2011;51(8):723–30. doi: 10.1080/10408391003785417 21838555

[4] Al-Eisawi DM. Flora of Jordan checklist. 1st ed. Amman: University of Jordan press; 2013.

[5] Oran SA. The status of medicinal plants in Jordan. J Agric Sci Technol A. 2014;4:461–7.

[6] Salim G, El-Dakdouki M, Abdallah H, Nasser H, Arnold-Apostolides N. Antioxidative and hepatoprotective effects of Rubus canescens DC. growing wild in Lebanon. Natural Product Journal. 2021;11:44–56.

[7] Altemimi A, Lakhssassi N, Baharlouei A, Watson DG, Lightfoot DA. Phytochemicals: Extraction, Isolation, and Identification of Bioactive Compounds from Plant Extracts. Plants (Basel). 2017;6(4):42. doi: 10.3390/plants6040042 28937585 PMC5750618

[8] Shishir MRI, Xie L, Sun C, Zheng X, Chen W. Advances in micro and nano-encapsulation of bioactive compounds using biopolymer and lipid-based transporters. Trends Food Sci Technol. 2018;78:34–60. doi: 10.1016/j.tifs.2018.05.018

[9] Mahmood TH, Al-Samydai A, Sulaibi MA, Alqaraleh M, Abed AI, Shalan N, et al. Development of Pegylated Nano-Phytosome Formulation with Oleuropein and Rutin to Compare Anti-Colonic Cancer Activity with Olea Europaea Leaves Extract. Chem Biodivers. 2023;20(8):e202300534. doi: 10.1002/cbdv.202300534 37498138

[10] Singh M, Devi S, Rana VS, Mishra BB, Kumar J, Ahluwalia V. Delivery of phytochemicals by liposome cargos: recent progress, challenges and opportunities. J Microencapsul. 2019;36(3):215–35. doi: 10.1080/02652048.2019.1617361 31092084

[11] Alshaikh F, Al-Samydai A, Issa R, Alshaer W, Alqaraleh M, Al-Halaseh LK, et al. Encapsulation of gingerol into nanoliposomes: Evaluation of in vitro anti-inflammatory and anti-cancer activity. Biomed Chromatogr. 2024;38(8):e5899. doi: 10.1002/bmc.5899 38797863

[12] Bourgaud F, Gravot A, Milesi S, Gontier E. Production of plant secondary metabolites: a historical perspective. Plant Science. 2001;161(5):839–51. doi: 10.1016/s0168-9452(01)00490-3

[13] Rauha JP. The search for biological activity in Finnish plant extracts containing phenolic compounds. Ph.D. Dissertation, University of Helsinki. 2001. Available from: https://helda.helsinki.fi/server/api/core/bitstreams/5444b1e4-12d9-4319-a98d-6b98fc9edc57/content

[14] Abu Shaqra QM. Epidemiological aspects of brucellosis in Jordan. Eur J Epidemiol. 2000;16(6):581–4. doi: 10.1023/a:1007688925027 11049102

[15] Annunziata F, Pinna C, Dallavalle S, Tamborini L, Pinto A. An Overview of Coumarin as a Versatile and Readily Accessible Scaffold with Broad-Ranging Biological Activities. Int J Mol Sci. 2020;21(13):4618. doi: 10.3390/ijms21134618 32610556 PMC7370201

[16] MA H, MI Y. Microbiological Quality of Soft White Cheese Produced Traditionally in Jordan. J Food Process Technol. 2017;8(12):706–12. doi: 10.4172/2157-7110.1000706

[17] Alqaraleh S, Mehyar G, Alqaraleh M, Awaisheh S, Rahahleh R. Antibacterial and antioxidant activities of extracts from selected wild plant species found in Jordan. Tropical Journal of Natural Product Research. 2023;7:2520–4.

[18] Pratima NA, Gadikar R. Liquid chromatography-mass spectrometry and its applications: a brief review. Arch Org Inorg Chem Sci. 2018;1: 26-34.

[19] Krieger S, Hayen H, Schmitz OJ. Quantification of coumarin in cinnamon and woodruff beverages using DIP-APCI-MS and LC-MS. Anal Bioanal Chem. 2013;405(25):8337–45. doi: 10.1007/s00216-013-7238-x 23912829

[20] Awaisheh SS. Efficacy of Fir and Qysoom essential oils, alone and in combination, in controlling Listeria monocytogenes in vitro and in RTE meat products model. Food Control. 2013;34: 657–661.

[21] Yasunaka K, Abe F, Nagayama A, Okabe H, Lozada-Pérez L, López-Villafranco E, et al. Antibacterial activity of crude extracts from Mexican medicinal plants and purified coumarins and xanthones. J Ethnopharmacol. 2005;97(2):293–9. doi: 10.1016/j.jep.2004.11.014 15707768

[22] Donsì F, Annunziata M, Sessa M, Ferrari G. Nanoencapsulation of essential oils to enhance their antimicrobial activity in foods. LWT - Food Science and Technology. 2011;44(9):1908–14. doi: 10.1016/j.lwt.2011.03.003

[23] Celeghini R, Vilegas J, Lanças F. Extraction and quantitative HPLC analysis of coumarin in hydroalcoholic extracts of Mikania glomerata Spreng: (“guaco”) leaves. J Braz Chem Soc. 2001;12:706–9.

[24] Maritim S, Boulas P, Lin Y. Comprehensive analysis of liposome formulation parameters and their influence on encapsulation, stability and drug release in glibenclamide liposomes. Int J Pharm. 2021;592 120051. doi: 10.1016/j.ijpharm.2020.120051 33161039

[25] Nsairat H, Khater D, Sayed U, Odeh F, Al Bawab A, Alshaer W. Liposomes: Structure, composition, types, and clinical applications. Heliyon. 2022;8(5):e09394–409. 35600452 10.1016/j.heliyon.2022.e09394 PMC9118483

[26] Woodle MC, Lasic DD. Sterically stabilized liposomes. Biochim Biophys Acta. 1992;1113(2):171–99. doi: 10.1016/0304-4157(92)90038-c 1510996

[27] Lin M, Qi XR. Purification method of drug-loaded liposome. In: Lu WL, Qi XR, editors. Liposome-based drug delivery systems. Berlin: Springer; 2021. pp. 111-121.

[28] Xu X, Khan MA, Burgess DJ. A two-stage reverse dialysis in vitro dissolution testing method for passive targeted liposomes. Int J Pharm. 2012;426(1–2):211–8. doi: 10.1016/j.ijpharm.2012.01.030 22301423

[29] Odeh F, Naffa R, Azzam H, Mahmoud IS, Alshaer W, Al Bawab A, et al. Co-encapsulation of thymoquinone with docetaxel enhances the encapsulation efficiency into PEGylated liposomes and the chemosensitivity of MCF7 breast cancer cells to docetaxel. Heliyon. 2019;5(11):e02919. doi: 10.1016/j.heliyon.2019.e02919 31844767 PMC6895652

[30] Youssef AM, El-Sayed SM, Salama HH, El-Sayed HS, Dufresne A. Evaluation of bionanocomposites as packaging material on properties of soft white cheese during storage period. Carbohydr Polym. 2015;132:274–85. doi: 10.1016/j.carbpol.2015.06.075 26256350

[31] Cui HY, Wu J, Lin L. Inhibitory effect of liposome-entrapped lemongrass oil on the growth of Listeria monocytogenes in cheese. J Dairy Sci. 2016;99(8):6097–104. doi: 10.3168/jds.2016-11133 27265173

[32] Jirovetz L, Buchbauer G, Stoilova I, Stoyanova A, Krastanov A, Schmidt E. Chemical composition and antioxidant properties of clove leaf essential oil. J Agric Food Chem. 2006;54(17):6303–7. doi: 10.1021/jf060608c 16910723

[33] Raters M, Matissek R. Analysis of coumarin in various foods using liquid chromatography with tandem mass spectrometric detection. Eur Food Res Technol. 2007;227(2):637–42. doi: 10.1007/s00217-007-0767-9

[34] Sproll C, Ruge W, Andlauer C, Godelmann R, Lachenmeier DW. HPLC analysis and safety assessment of coumarin in foods. Food Chem. 2008;109(2):462–9. doi: 10.1016/j.foodchem.2007.12.068 26003373

[35] Assafiri O, Abdallah H, El-Dakdouki M. Antibacterial effect and phytochemical analysis of the shoot system of rubus canescens dc. Growing in lebanon. BAU Journal - Science and Technology. 2020;2(1):. doi: 10.54729/2706-784x.1050

[36] Martini S, D’Addario C, Colacevich A, Focardi S, Borghini F, Santucci A, et al. Antimicrobial activity against Helicobacter pylori strains and antioxidant properties of blackberry leaves (Rubus ulmifolius) and isolated compounds. Int J Antimicrob Agents. 2009;34(1):50–9. doi: 10.1016/j.ijantimicag.2009.01.010 19386474

[37] Zhang H-Y, Wang L-F. Theoretical elucidation of structure–activity relationship for coumarins to scavenge peroxyl radical. Journal of Molecular Structure: THEOCHEM. 2004;673(1–3):199–202. doi: 10.1016/j.theochem.2003.12.014

[38] Kostova I, Bhatia S, Grigorov P, Balkansky S, Parmar VS, Prasad AK, et al. Coumarins as antioxidants. Curr Med Chem. 2011;18(25):3929–51. doi: 10.2174/092986711803414395 21824098

[39] Liang C-H, Chou T-H. Effect of chain length on physicochemical properties and cytotoxicity of cationic vesicles composed of phosphatidylcholines and dialkyldimethylammonium bromides. Chem Phys Lipids. 2009;158(2):81–90. doi: 10.1016/j.chemphyslip.2009.01.006 19428352

[40] Chen M, Fang X, Tang S, Zheng N. Polypyrrole nanoparticles for high-performance in vivo near-infrared photothermal cancer therapy. Chem Commun (Camb). 2012;48(71):8934–6. doi: 10.1039/c2cc34463g 22847451

[41] Khan J, Alexander A, , Saraf S, Saraf S. Luteolin-phospholipid complex: preparation, characterization and biological evaluation. J Pharm Pharmacol. 2014;66(10):1451–62. doi: 10.1111/jphp.12280 24934881

[42] Danaei M, Dehghankhold M, Ataei S, Hasanzadeh Davarani F, Javanmard R, Dokhani A, et al. Impact of Particle Size and Polydispersity Index on the Clinical Applications of Lipidic Nanocarrier Systems. Pharmaceutics. 2018;10(2):57. doi: 10.3390/pharmaceutics10020057 29783687 PMC6027495

[43] Teulon J-M, Godon C, Chantalat L, Moriscot C, Cambedouzou J, Odorico M, et al. On the Operational Aspects of Measuring Nanoparticle Sizes. Nanomaterials (Basel). 2018;9(1):18. doi: 10.3390/nano9010018 30583592 PMC6359205

[44] Al-Samydai A, Abu Hajleh MN, Al-Sahlawi F, Nsairat H, Khatib AA, Alqaraleh M, et al. Advancements of metallic nanoparticles: A promising frontier in cancer treatment. Sci Prog. 2024;107(4):368504241274967. doi: 10.1177/00368504241274967 39370817 PMC11459474

[45] Alqarni MH, Foudah AI, Alam A, Salkini MA, Muharram MM, Labrou NE, et al. Coumarin-Encapsulated Solid Lipid Nanoparticles as an Effective Therapy against Methicillin-Resistant Staphylococcus aureus. Bioengineering (Basel). 2022;9(10):484. doi: 10.3390/bioengineering9100484 36290453 PMC9598203

[46] Lowry GV, Hill RJ, Harper S, Rawle AF, Hendren CO, Klaessig F, et al. Guidance to improve the scientific value of zeta-potential measurements in nanoEHS. Environ Sci: Nano. 2016;3(5):953–65. doi: 10.1039/c6en00136j

[47] Srinath P, Vyas SP, Diwan PV. Preparation and pharmacodynamic evaluation of liposomes of indomethacin. Drug Dev Ind Pharm. 2000;26(3):313–21. doi: 10.1081/ddc-100100359 10738648

[48] Thakur S, Ray S, Jhunjhunwala S, Nandi D. Insights into coumarin-mediated inhibition of biofilm formation in Salmonella Typhimurium. Biofouling. 2020;36(4):479–91. doi: 10.1080/08927014.2020.1773447 32546074

[49] Reen FJ, Gutiérrez-Barranquero JA, Parages ML, O Gara F. Coumarin: a novel player in microbial quorum sensing and biofilm formation inhibition. Appl Microbiol Biotechnol. 2018;102(5):2063–73. doi: 10.1007/s00253-018-8787-x 29392389 PMC5814477

[50] Stan D, Enciu A-M, Mateescu AL, Ion AC, Brezeanu AC, Stan D, et al. Natural Compounds With Antimicrobial and Antiviral Effect and Nanocarriers Used for Their Transportation. Front Pharmacol. 2021;12:723233. doi: 10.3389/fphar.2021.723233 34552489 PMC8450524

[51] Nitiema L, Savadogo A, Simpore J, Dianou D, Traore A. In vitro antimicrobial activity of some phenolic compounds (coumarin and quercetin) against gastroenteritis bacterial strains. Int J Microbiol Res. 2012;3:183–7.

[52] da Cunha MG, de Cássia Orlandi Sardi J, Freires IA, Franchin M, Rosalen PL. Antimicrobial, anti-adherence and antibiofilm activity against Staphylococcus aureus of a 4-phenyl coumarin derivative isolated from Brazilian geopropolis. Microb Pathog. 2020;139:103855. doi: 10.1016/j.micpath.2019.103855 31706001

[53] Tannous RI. Miscellaneous white brined cheeses. In: Robinson K, Tamime AY, editors. Feta and related cheeses. Cambridge: Wood Head Publishing Limited; 1991. p. 208–228.

